# An evidence-based gamified mHealth intervention for overweight young adults with maladaptive eating habits: study protocol for a randomized controlled trial

**DOI:** 10.1186/s13063-017-2340-6

**Published:** 2017-12-12

**Authors:** Ioana R. Podina, Liviu A. Fodor, Ana Cosmoiu, Rareș Boian

**Affiliations:** 1 Laboratory of Cognitive Clinical Sciences, Department of Psychology, University of Bucharest, 90 Panduri Street, Bucharest, 050657 Romania; 2 International Institute for The Advanced Studies of Psychotherapy and Applied Mental Health, Babeș-Bolyai University, 37 Republicii Street, Cluj-Napoca, 400015 Romania; 3 Evidence-Based Psychological Assessment and Interventions Doctoral School, Babeș-Bolyai University, 37 Republicii Street, Cluj-Napoca, 400015 Romania; 4 Department of Computer Science, Babeş-Bolyai University, Mihail Kogălniceanu Street, Cluj-Napoca, 400084 Romania

**Keywords:** mHealth, CBT, Maladaptive, Gamification, Overweight, Young adults

## Abstract

**Background:**

Cognitive behavior therapy (CBT) is the first-line of treatment for overweight and obesity patients whose problems originate in maladaptive eating habits (e.g., emotional eating). However, in-person CBT is currently difficult to access by large segments of the population. The proposed SIGMA intervention (i.e., the Self-help, Integrated, and Gamified Mobile-phone Application) is a mHealth intervention based on CBT principles. It specifically targets overweight young adults with underlying maladaptive behaviors and cognitions regarding food. The SIGMA app was designed as a serious game and intended to work as a standalone app for weight maintenance or alongside a calorie-restrictive diet for weight loss. It uses a complex and novel scoring system that allows points earned within the game to be supplemented by points earned during outdoor activities with the help of an embedded pedometer.

**Methods/design:**

The efficacy of the SIGMA mHealth intervention will be investigated within a randomized, placebo-controlled trial. The intervention will be set to last 2 months with a 3-month follow-up. Selected participants will be young overweight adults with non-clinical maladaptive eating habits embodied by food cravings, binge eating, and emotional eating. The primary outcomes will be represented by changes in (1) self-reported maladaptive thoughts related to eating and body weight, (2) self-reported maladaptive eating behaviors in the range of urgent food cravings, emotional eating or binge eating, (3) as well as biased attentional processing of food items as indexed by reaction times. Secondary outcomes will be represented by changes in weight, Body Mass Index, general mood, and physical activity as indexed by the number of steps per day.

**Discussion:**

Through an evidence-based cognitive behavioral approach and a user-friendly game interface, the SIGMA intervention offers a significant contribution to the development of a cost-effective and preventive self-help tool for young overweight adults with maladaptive eating habits.

**Trial registration:**

ISRCTN, ID: 70907354. Registered on 6 February 2017. The ISRCTN registration is in line with the World Health Organization Trial Registration Data Set. The present paper represents the original version of the protocol. Any changes to the protocol will be communicated to ISRCTN.

**Electronic supplementary material:**

The online version of this article (doi:10.1186/s13063-017-2340-6) contains supplementary material, which is available to authorized users.

## Background

Obesity, commonly characterized by a Body mass Index (BMI) equal to or exceeding 30 kg/m^2^, has become a worldwide health issue with consequences such as morbidity, disability, chronic diseases, and emotional health problems associated with weight stigma [1, 2]. Currently, obesity and overweight affect as many as 30% of the worldwide population and this number is expected to grow up to 50% by 2030 [3]. The high rates of obesity and their aforementioned health consequences strain the public health system and result in significant economic and societal burden [4]. This highlights an urgent need for readily accessible evidence-based interventions aimed at prompting weight loss and weight maintenance.

Currently, CBT is the first-line of treatment for overweight and obesity cases that originate in *maladaptive eating habits* (i.e., eating in the absence of hunger, eating prompted by stress or negative emotionality) [5, 6]. Maladaptive eating habits are the main cause behind “yo-yo” dieting and represent a barrier to losing weight and to a healthy lifestyle [7], as well as an important relapse factor after bariatric surgery [8].

CBT targets not only (1) maladaptive behavioral habits, but also (2) *maladaptive cognitive styles* (e.g., dysfunctional or unhealthy beliefs). Maladaptive cognitive styles are central in CBT and are theorized to underlie negative emotions and undesirable behaviors, such as emotional eating, as indicated by several trials [9, 10] and reviews (e.g., [11]).

Maladaptive behaviors and cognitive styles can be best assessed and altered in their ecological environment. A potential avenue towards achieving this is the delivery of interventions through smartphone apps (i.e., mHealth interventions). These interventions are particularly relevant for the young adult population (i.e., 18 to 35 years old) for two main reasons. Firstly, young adults (i.e., 18 to 35 years old) are particularly susceptible to becoming overweight or obese (e.g., [12]). Moreover, weight gain during this life-stage is not only a marker of obesity but also for developing chronic disease risk factors (e.g., high blood pressure; [13]). Secondly, young adults are the most likely age group to own and constantly interact with smartphones. As many as 100% of young adults in developed countries own smartphones, with constantly increasing rates in developing countries as well [14, 15]. Therefore, the increased usage of mobile phones among the young adult population and the health consequences associated with early-life weight gain provide the motivation for delivering weight management interventions (i.e., mHealth) on a large scale and in an ecological manner.

Mobile or mHealth interventions for weight management have demonstrated promising results across various studies [16]. However, they face two important limitations, as argued below.

The first limitation is that most studies report up to a 50% dropout rate in the use of existing mHealth and eHealth applications (i.e., electronic/technologically mediated apps), which makes them subject to short-term use only [17]. A potential solution for long-term use would be to increase the interactivity and attraction of current mHealth interventions via gamification. Gamification refers to the employment of game-like components (e.g., challenges, storylines) in non-game contexts such as psychological interventions [18]. Although research on the topic remains in its initial stages, current evidence suggests that gamification can have a positive impact on motivation and health behaviors [19] and, most importantly, it promotes long-term treatment adherence [18, 20, 21].

A second limitation of existing m/eHealth interventions is that currently there is no available application or scientific trial targeting maladaptive eating habits despite their high prevalence in overweight individuals [22]. Hence, new portable, evidence-based, integrated, and interactive applications for weight management are needed.

Therefore, the purpose of the SIGMA application (i.e., the Self-help, Integrated, and Gamified Mobile-phone Application) is to primarily address the maladaptive behavioral and cognitive styles that impede weight management in young adults at risk for obesity (BMI 25–29.9 kg/m^2^). The SIGMA app is a CBT-based intervention that was designed as a serious game and is intended to work as a standalone app for weight maintenance or alongside a calorie-restrictive diet for weight loss. The aim of this report is to describe the theoretical rationale and intervention design of the SIGMA study.

### Theoretical framework

The SIGMA intervention was informed by CBT’s *cognitive ABC model* (*A*ntecedents – *B*eliefs – *C*onsequences). The cognitive ABC model states that negative emotions and undesirable eating behaviors (C) are caused and maintained, contrary to common beliefs, not by adversities or antecedents (A), but by (1) maladaptive beliefs and (2) faulty information processing (B) [23] concerning those adversities, as evidenced below.

1. Maladaptive beliefs are central in CBT for obesity. Prior research has indicated that obese participants have more unhealthy food and weight-related beliefs including catastrophizing, faulty body-image perception, and poor self-control than healthy-weight participants [24–26]. According to CBT’s ABC model, maladaptive beliefs (B), particularly sabotaging thoughts, cause uncontrolled and unplanned eating (C). Osberg, Poland, Aguayo, and MacDougall ([27], p.26) define sabotaging thoughts as “cognitively distorted and unhealthy attitudes and beliefs regarding food” (e.g., I can’t possibly live without chocolate).
  An example of the cognitive ABC model is the following: (A) a feeling of sadness in the context of losing a job triggers a sabotaging thought (B) – “I hate this feeling. If I eat I will feel better” that causes (C) emotional eating followed by guilt (A) and the loop of maladaptive cognitive and behavioral styles is preserved. Hence, CBT’s *mechanism of change* is not A, nor C, but B. In other words, the aim of CBT is to replace maladaptive beliefs with more adaptive and healthy alternatives (e.g., “I don’t like this feeling, but eating won’t solve my problem”) that help adherence to a calorie-restricted diet and prevent gaining any additional weight [28]
2. Another central factor in the CBT conceptualization of obesity refers to faulty information processing, a selective processing of food stimuli in the environment. One highly investigated faulty information process is attention bias to food cues, otherwise known as the tendency to attend to food stimuli. A growing body of research has indicated that biased attention toward food predicts the strength of cravings [29], stress eating [30], the amount eaten, and even the amount of weight gained in obesity cases [31, 32]

Attention bias is theorized to precede maladaptive beliefs, making environmental stimuli more difficult to resist [33]. Targeting attention biases is a complementary path that maximizes resistance to tempting situations which become less likely to trigger sabotaging thoughts. This process operates at an implicit level, but there is evidence that it can be modified by specific interventions (i.e., ABM – attention bias modification; [34]) that can be successfully integrated into CBT [35].

Overall, the ABC model provides an evidence-based theoretical guiding structure for the SIGMA intervention.

## Methods/design

The SIGMA trial will be a randomized, placebo-controlled trial designed to last for a total of 22 weeks including a 2-week baseline point. The randomization procedure is described in detail in the “Randomization and blinding” section. The primary objective of this trial is to contrast the SIGMA intervention against a sham intervention that will include all the modules developed for the SIGMA intervention with the exception of the gamified intervention module. Therefore, it will lack the active/distinctive features of the SIGMA app.

The Standard Protocol Items Recommendations for Interventional Trials (SPIRIT) Statement 2013 are followed (see also Additional file 1: SPIRIT 2013 Checklist: recommended items to address in a clinical trial protocol and related documents). The trial was registered under the registration number ISRCTN70907354 on 6 February 2017.

### Intervention design

**Fig. 1:**
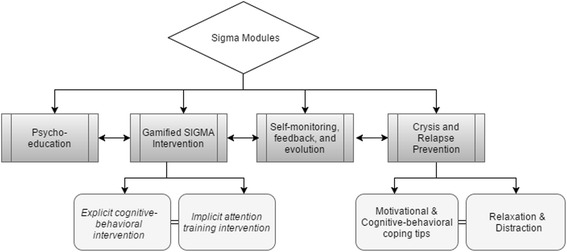
Overview of the Self-help, Integrated, and Gamified Mobile-phone Application (SIGMA) trial configuration

The SIGMA intervention is designed to accommodate four mHealth modules, which will be further described in the following paragraphs and are depicted in Fig. 1. For the purposes of the SIGMA trial, all participants (both intervention and control groups) will be asked to actively follow a calorie-restrictive diet of their own choice. General information about dieting and daily exercising is embedded in the psycho-education module of the SIGMA app and should assist in choosing a healthy and balanced calorie-restrictive diet. This information will also be accessible online via the study’s dedicated website if more detailed content is needed.

#### The psycho-education module

On opening the app, users are prompted to access the psycho-education module where information about the purpose of the app, as well as information about physical activity and dieting is provided. Users are informed about the etiological role played by maladaptive behavioral and cognitive styles in gaining weight. Other included aspects are the differences between hunger and craving and information on how controlling the environment (e.g., not having tempting food in the house) may be a successful strategy for weight management. Moreover, the psycho-educational content will also be present throughout the app’s usage in the form of daily tips and messages.

#### The gamified intervention module

The gamified intervention module incorporates two sub-modules, namely the explicit cognitive-behavioral intervention and the implicit attention-training intervention, which are described below. The SIGMA modules, especially the intervention module, follow the guidelines of the Beck CBT protocol for weight management [36].

#### The explicit cognitive-behavioral intervention (SIGMAe)

**Fig. 2:**
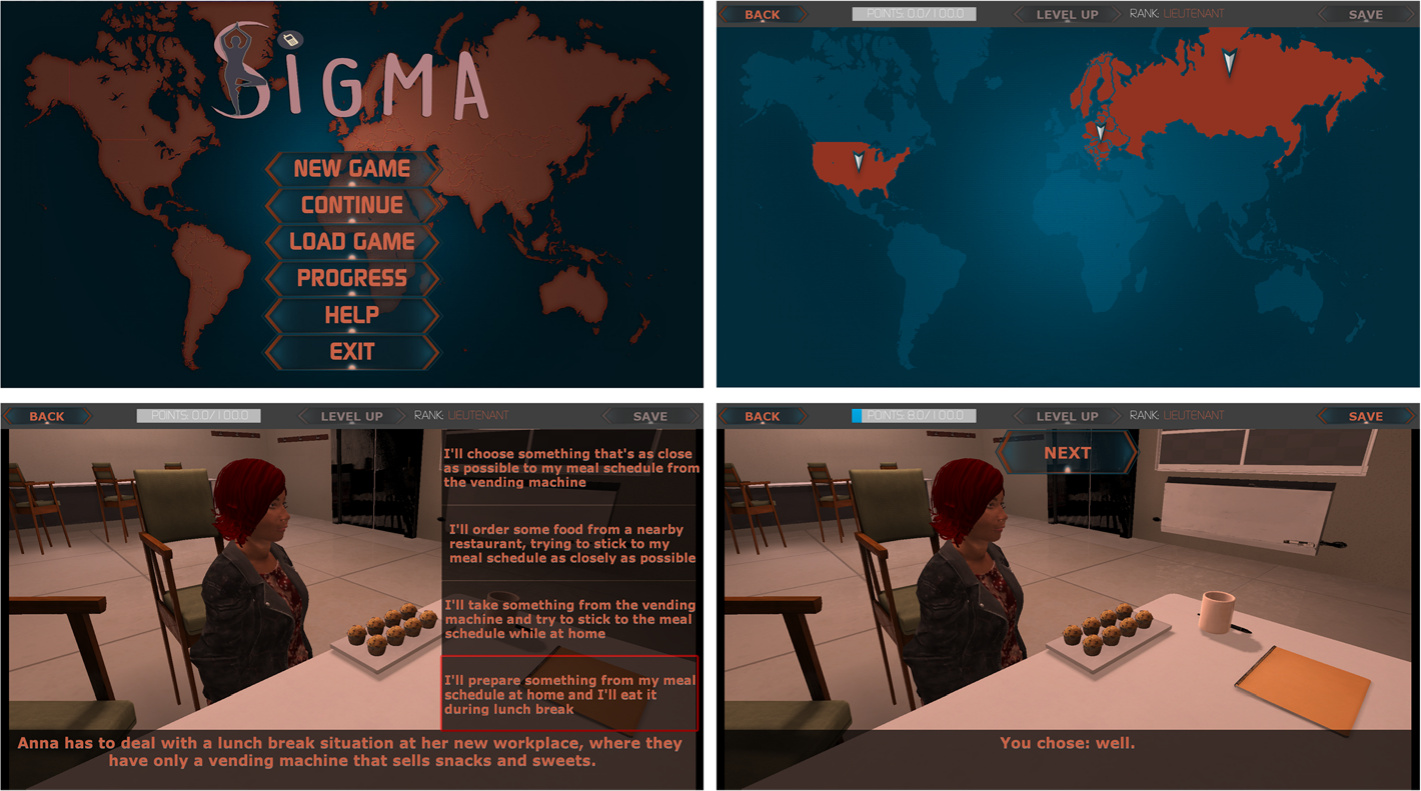
Overview of the Self-help, Integrated, and Gamified Mobile-phone Application - explicit cognitive-behavioral intervention (SIGMAe) interface

This component of the intervention targets sabotaging thoughts regarding food, as well as maladaptive eating habits (Fig. 2). The gamified interface is an important element of this module, providing (1) a storyline, (2) animated characters that go through difficult and tempting situations, (3) learning opportunities to cope with temptations, and (4) a reward point system that opens new theory-based game levels, as detailed below. The SIGMAe game is inspired by the Beck CBT protocol for weight management [36].

The storyline is standard; the users learn that they are superheroes in training who should help save the world and the characters from eating temptations. In the game, the characters find themselves in a situation where they should resist temptations, such as eating some highly palatable food, eating when feeling distressed or eating in a social context. To facilitate real-life application, the game-settings are diverse, varying from home-inspired scenarios to holiday and social gathering scenarios. The user’s task is to assist the characters in making a decision in the context of a problematic situation. This will be achieved by choosing the best coping card out of four possible alternatives, varying from the worst coping option to the best coping option (Table 1). These alternatives are organized by levels and can target either behavioral choices, cognitive self-statements or a combination of both (Table 1). Once the user has chosen a coping card, SIGMA will provide *healthy habit points*, which increase the user’s total score and *mastery* level and allow them to further advance in the game. Noteworthy, the app provides feedback, explaining why the chosen coping card is correct or not.

**Table 1:** The Self-help, Integrated, and Gamified Mobile-phone Application explicit cognitive-behavioral intervention (SIGMAe) behavioral (B), cognitive (C), and cognitive-behavioral (CB) coping choices exemplified

Worst coping card alternative		Best coping card alternative
Emotional eating scenario: Ann just got separated from her boyfriend. She is sad and in order to help her feel better her friends booked a table at her favorite restaurant. Ann says to herself:		
B	I can hardly wait to go to the restaurant and eat all I can eat to feel better	I know that my favorite restaurant will be a tempting setting for me, especially in these circumstances. I suggest going to bowling
C	It’s terrible what happened to me. There is no way out. My friends are right; we should go out and eat	It is unpleasant what happened to me; however, I can cope with this situation
CB		It is unpleasant what happened to me; however, I can cope with this situation. In addition, there are plenty more things to do than eating my feelings, like go bowling
Binge-eating scenario: Eliza is approaching the fridge. She feels like she is going to lose control. Eliza says to herself:		
B	I can’t help myself. I will eat as much as I want	I will do my relaxation exercises and then I’ll read something
C	It is not fair. Others can eat all they want. Why shouldn’t I?	It may not seem fair; however, eating until I can no longer eat is hardly a solution
CB		It may not seem fair; however, eating until I can no longer eat is hardly a solution. Instead, I will do my relaxation exercises
Craving scenario: when watching TV, Daniel is always tempted to eat a bag of chips or chew something. He says:		
B	I’ll eat chips while watching TV	If I am going to watch TV, I might as well play with my dog
C	I must eat something; I am not used to simply watching TV	No need to eat chips while I watch TV. I can do without
CB		No need to eat chips while I watch TV. I can do without or if not I can play with my dog

The complexity of the explicit intervention will increase as the user interacts with the app and accumulates more points. The tasks at hand provide three levels of difficulty as follows: easy (behavioral), medium (cognitive), and complex (cognitive-behavioral) (Table 1). In order to facilitate learning, previously encountered scenarios at each level will be repeated in a random fashion. The application offers the option of social media sharing as well.

The gamified intervention consists of 300 scenarios including craving, binge, and emotional eating scenarios. In order to ensure a steady progression and involvement, the user will be limited to solving a fixed number of scenarios per week. Given that the intervention protocol is set to extend over 2 months (8 weeks), a number of 37 scenarios per week are to be solved in order for all the behavioral, cognitive, and cognitive-behavioral scenarios to be addressed.

#### The implicit attention-training intervention (SIGMAi)

**Fig. 3:**
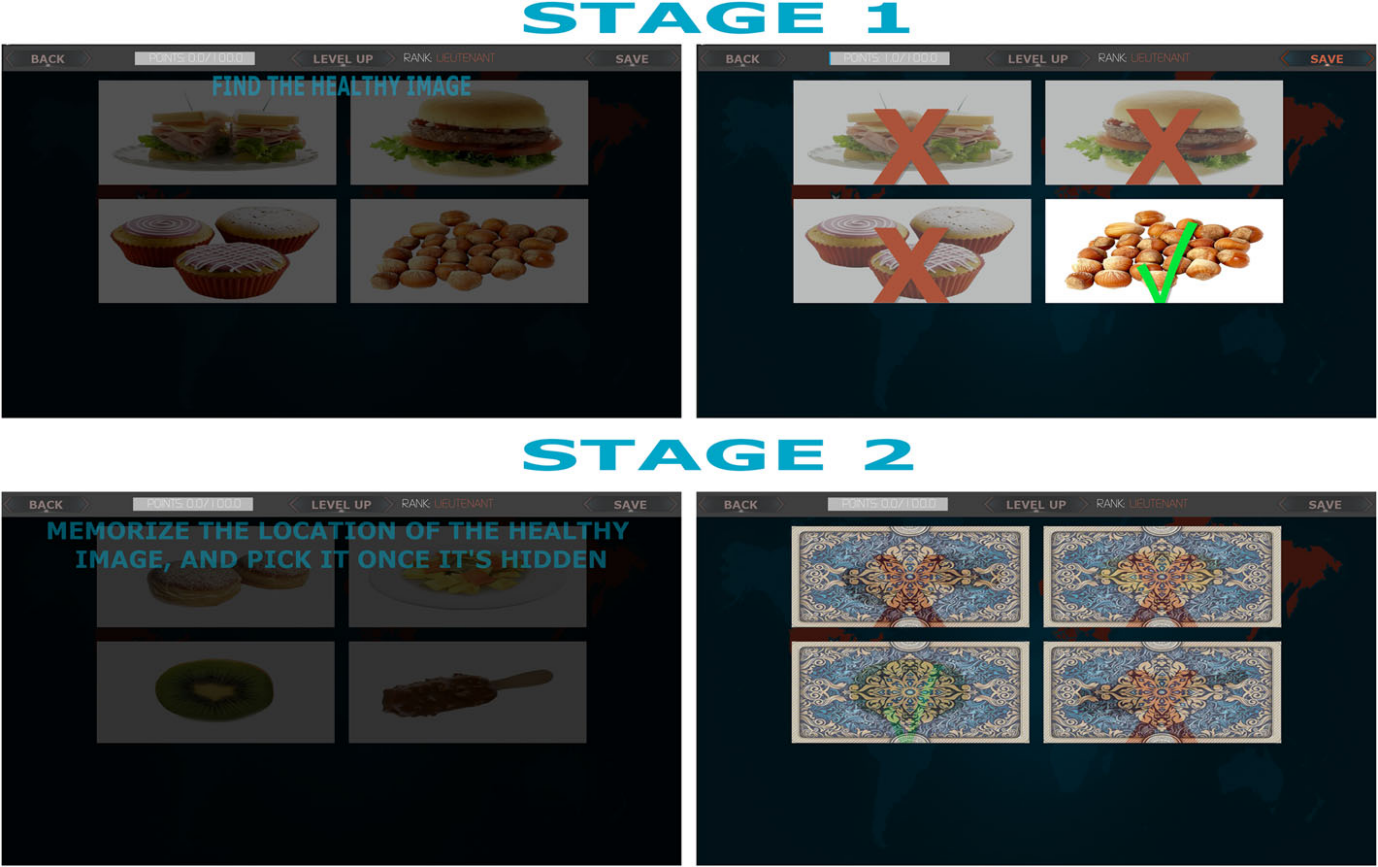
Overview of the Self-help, Integrated, and Gamified Mobile-phone Application - implicit attention-training intervention (SIGMAi) interface

The implicit component of the gamified intervention is aimed at addressing the biased attention towards appetizing stimuli. Therefore, SIGMAi trains the user’s implicit attentional processes towards healthy food choices, while redirecting them from the unhealthy ones. This intervention is inspired by attention bias modification procedures [37] and has two main levels described below, and graphically depicted in Fig. 3.

Within the first level, a minimum of two and a maximum of six food images appear simultaneously on the screen while only one food image represents a healthy choice. Its location on the screen varies randomly with each trial. The participant has to choose the healthy food item as fast as possible while ignoring the unhealthy and possibly more appetizing ones. If no choice is made within 2500 ms, the task moves on to the next trial. The second level of gameplay will present the user with two up to six food items for a short amount of time varying from 700 ms to 1200 ms and depending on the number of images per trial. Once the allocated time has expired, the images will be flipped showing a non-descript reverse side. The user will have to remember the location of the healthy food item and flip the reverse side of the correct image.

As in the case of SIGMAe, in order to assure a steady progression and involvement, the user will be limited to solving a fixed number of trials per week. Overall, the intervention protocol is set to accommodate 975 weekly trials and a total of 7800 trials within the 2 months of the intervention.

The stimuli employed in the attention-training intervention were collected from Food-pics ([38]), a database consisting of standardized images of food stimuli. With each healthy food choice, the user earns one healthy habit point. Hence, due to the design and reward points system, the SIGMAi intervention not only helps the user learn the distinction between healthy and unhealthy food items, but it also makes unhealthy food choices less salient [39]. As in the case of SIGMAe, the results of the game can also be shared on social media and with other users.

#### The Crisis and Relapse prevention module

An important component of SIGMA, previously unaddressed in eHealth interventions, is the presence of a *crisis intervention module*. The need for such a module becomes apparent as relapses in dieting are more likely to occur in moments of crisis (e.g., cravings, a decreased mood) [40, 41]. SIGMA’s crisis intervention module is specifically tailored to address these situations. The features of the crisis module are described below.

#### Motivational messages and coping strategies

The app offers, when requested, written motivational messages or cognitive-behavioral coping strategies, mimicking the coping tips offered by SIGMAe and dependent on the type of encountered issues (e.g., craving, boredom, stress or low mood).

#### Relaxation tools

In the context of emotional eating, particularly for those users who are vulnerable to temptation under stressful conditions, SIGMA’s crisis module will provide guidance in performing relaxing breathing exercises. This is achieved by way of a visual breathing aid. The user can choose between a predefined and a customized breathing rhythm. As a visual aid, an onscreen balloon will expand or contract following the chosen calming breathing pace.

#### Distraction

Because it mimics a standard game (e.g., fast responses to challenges, shifting stimuli) and because it relies on fast and effortlessly responses, the SIGMAi module can also be used for distraction purposes via the crisis module.

#### The self-monitoring, feedback, and evolution module

Only the design and key features of the module will be discussed here, details on the instruments used for self-monitoring are described in the “Outcomes” section of the paper.

#### Self-monitoring

The self-monitoring module serves two relevant functions, detailed below.

The first function is to assist users in self-monitoring their own eating and physical activity patterns, a proven predictor of weight-loss and weight management [42]. SIGMA includes self-monitoring components aimed at monitoring dietary intake and physical activity, enabling users to plan a meal/menu and physical exercise in advance and offering personalized tips (i.e., psycho-educational content) and feedback regarding eating and physical activity styles.

Aside from planning, the monitoring of physical activity is aided by an embedded *pedometer*. The decision to incorporate a pedometer was informed by the fact that it has been reliably associated with significant increases in physical activity and significant decreases in BMI ([43]). The pedometer will monitor and compare the user’s daily performance with a daily suggested target and will provide feedback and healthy habit points accordingly. As such, a norm of between 5000 and 7499 steps/day is considered low active, 7500 to 9999 steps/day is somewhat active, and 10,000 or more steps/day is considered an active lifestyle [44]).

The second function, drawing on the cognitive-behavioral principles, is to monitor how well the participants apply the CBT principles of the SIGMA game to real-life situations. Hence, a special feature of the monitoring module is the *ABC diary* that focuses on understanding the *A*ntecedents and *C*onsequences of maladaptive *B*eliefs regarding food, weight or the ability to maintain the diet. This should aid the users in noticing and challenging their maladaptive patterns without relying on a therapist for weight management.

Regarding the consequences of eating behaviors, users will be enabled to monitor their levels of reported satiety after a meal, as well as their emotional reactions after eating, such as guilt or satisfaction. Lower levels of satiety and negative emotional reactions after eating may predict a relapse and it is important for the app to offer alternative ways of thinking in tempting or adverse situations. This should help alleviate urges to eat outside planned meals or prevent feelings of guilt when it happens.

#### Feedback and evolution

The first 2 weeks of SIGMA usage focus on calibrating the SIGMA intervention through a baseline evaluation of the users’ behavioral and cognitive patterns (e.g., more emotional eating content for individuals with emotional eating issues). Following the initial baseline evaluation, the SIGMA app will produce a report identifying the users’ vulnerabilities that will also serve as a starting point for customizing the intervention. For instance, if the evaluation reveals that a user is more likely to succumb to dietary temptations in the evening, the app will send more tips and motivational messages at that specific time. Moreover, the feedback report is designed to be intuitive; for instance, the user’s points and the number of steps on the pedometer are delivered through a meaningful interpretation of the progress (e.g., “This week, you have accumulated X out of Y possible points and the total number of steps taken is equivalent to the distance from A to B”).

Feedback plays an important role in the serious game module, as each progress or failure is followed by feedback along with a detailed statement explaining why the specific choice made during SIGMAe or SIGMAi was erroneous. SIGMA will also provide feedback, in the form of charts, regarding the cognitive, behavioral, and emotional indexes of progress as compared to the user’s baseline level. Feedback on user’s progress (i.e., number of accumulated points, mastery level) will be based on objective assessments. These assessments are represented by both the healthy choices made during the SIGMAi trials, as well as the healthy cognitive and behavioral coping choices made during the SIGMAe trials.

Moreover, the SIGMA app uses a complex and novel scoring system that allows SIGMAe and SIGMAi points to be supplemented by points earned during outdoor activities with the help of a pedometer. As such, additional points are earned depending on the level of activity (i.e., daily step count) the user is willing to make. We do not want to encourage a fixation on other outcomes such as calorie counting or daily weighing [45]. However, we have embedded a calorie counter in the SIGMA app. Overall, all the earned points help the user reach a higher mastery level.

### The SIGMA randomized controlled trial (RCT)

The SIGMA trial is set to be a randomized, placebo-controlled trial that is nationally funded through a research grant. Throughout this trial, the SIGMA intervention will be contrasted against a specific form of placebo, also known as an *attention placebo control condition*. An attention placebo control refers to a condition that mimics an intervention but does not address the proposed mechanisms of change. Participants allocated to the attention placebo control condition will have full access to a modified version of the SIGMA app, which includes all the SIGMA modules except for the gamified intervention module. This control condition is highly suitable to investigate the active ingredients of an intervention, as in our case. Furthermore, this type of control arm is considered a highly valid control condition for RCT’s [46]. Ethical approval for this study was sought and received from the Ethics Committee of the Babeș-Bolyai University (Cluj-Napoca, Romania) and from the Ethics Committee of the University of Bucharest (Bucharest, Romania).

**Fig. 4:**
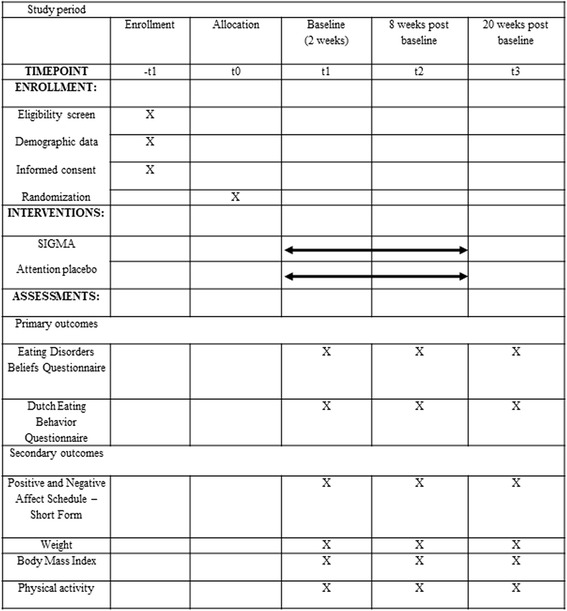
Recommendations for Interventional Trials (SPIRIT) figure – schedule of enrolment, intervention, and assessments

The SIGMA trial will have 2-week calibration point followed by a 2-month intervention and a 3-month follow-up. The schedule of the trial is presented Fig. 4.

The primary aim is to determine whether the SIGMA intervention is more effective than the attention placebo control condition in reducing maladaptive behaviors and cognitive styles, as well as in increasing their adaptive/functional counterparts. Our working hypothesis is that the SIGMA intervention will be significantly more effective in promoting change in maladaptive behaviors and cognitive styles, decreasing the maladaptive counterparts and increasing adaptive food-related behavioral and cognitive styles of response. Significant differences favoring the SIGMA intervention are to be expected in an evidence-based intervention, as more extensive use of theory in eHealth interventions is associated with an increase in effect size [47]. This change is expected to be maintained at follow-up.

The secondary aim is to determine whether the SIGMA intervention is more effective than the attention placebo control condition in reducing weight (e.g., kg, BMI), physical activity-related parameters (i.e., increase the number of steps per day), and general mood. The hypothesis is that the SIGMA intervention will be significantly more effective in prompting change in weight-related, physical activity patterns and even general mood relative to the attention placebo control group. This change is expected to be maintained at follow-up.

### Participants

#### Inclusion and exclusion criteria

Participants will be (1) young overweight adults (25 ≤ BMI ≤ 29.9), (2) aged between 18 and 35 years old, and with (3) maladaptive eating habits in the range of urgent food cravings, emotional eating or binge-eating patterns that do not meet the criteria for clinical eating disorders. Eligible participants will also have to own an Android-compatible smartphone that is able to connect to the Internet.

Volunteers will be excluded from the SIGMA trial in the following cases: (a) presence of any medical condition incompatible with physical/dietary recommendations (including pregnancy and type 2 diabetes); (b) presence of an eating disorder; (c) use of appetite-suppressing medication and/or current enrollment in other weight management programs; (d) current depression or any form of psychotic disorder; and (e) lack of access to an Android-compatible smartphone.

#### Recruitment

Potential participants will be recruited using multiple avenues of communication. Posters describing the intervention and the invitation to take part in the study will be posted in universities around the country. The electronic version of these posters will be distributed around the Internet with a special focus on weight-related forums, Facebook groups, and websites.

Emails describing the purpose of the intervention and an invitation to collaborate will be sent to entities concerned with curbing overweight/obesity rates. These are foundations or associations that are actively engaged in promoting healthy lifestyles by informing the general public about weight-related issues (e.g., Wings Foundation, The Association for Supporting Patients with Obesity, The Romanian Society for the Study of Obesity, etc.).

#### Sample size

In order to detect a medium effect size (i.e., Cohen’s *d* = 0.50), with a *p* ≤ 0.05 and 80% power, we would need a total of 74 participants, 37 participants per arm (calculated using G*Power, [48]). Assuming that up to 40% of participants will drop out of the intervention [16], a total of 104 participants will be needed (52 participants per trial arm) in order to detect the aforementioned effect size. The 40% percentage dropout rate represents the worst-case scenario as a high dropout rate can be sometimes expected in mHealth or eHealth studies [16].

#### Randomization and blinding

**Fig. 5:**
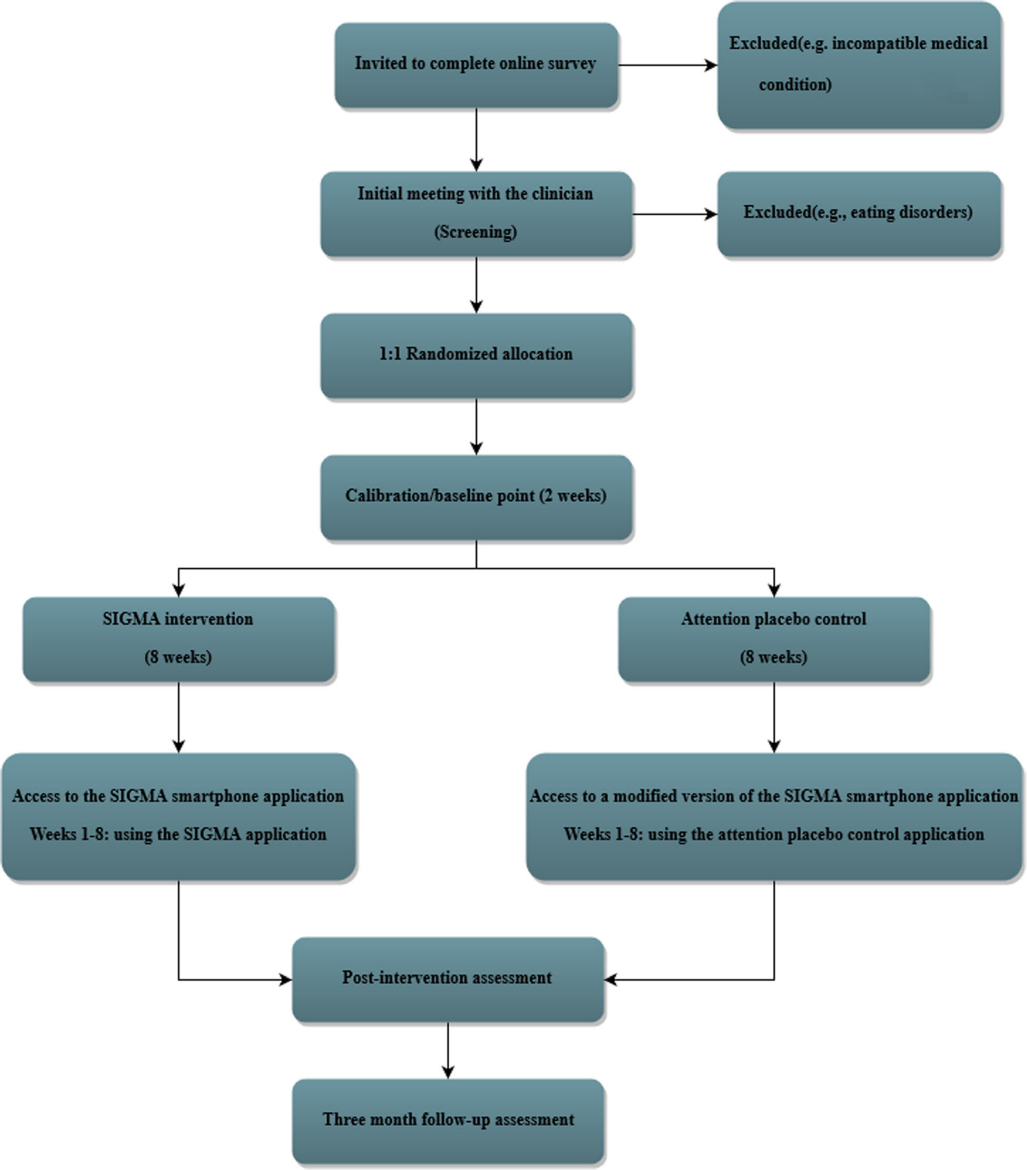
Self-help, Integrated, and Gamified Mobile-phone Application (SIGMA) study flow diagram

Selected participants will be randomized in the intervention and control trial arms in a 1:1 ratio (Fig. 5). An independent researcher will handle the randomization and the random sequence will be generated using a 1:1 allocation ratio via an online available random number generator (i.e., https://www.random.org/). More specifically, randomization will be prestratified by gender and follow a permuted-block randomization scheme to ensure a balance between the arms of the trial.

The randomization sequence will be concealed from the staff responsible with enrolling and assigning the participants in the trial arms. This objective will be achieved by using sealed envelopes that will be numbered in advance and opened sequentially only after the participant’s name will be written on the envelope. Similarly, the personnel responsible for analyzing the data will be blinded to participant allocation.

In addition, participants will be blinded to the nature of the group to which they will be assigned, but they will be informed that they have a 1 in 2 chance of being assigned to the placebo group. Given that we use an attention placebo control group that mimics the SIGMA intervention, we are confident in a successful blinding process of the participants.

### Outcome measures and evaluation instruments used outside the application

The evaluation of the primary and secondary outcomes will be conducted before the intervention (T0), at post intervention (T1 – after 2 months), and at follow-up (T2 – after 3 months of follow-up). There will also be a constant monitoring of some of the parameters, which will be described in detail below. Most self-reported measurements, except for weight and BMI, will be recorded through the study’s website to ease visibility of multi-item questionnaires. The remaining variables, such as daily steps, attention bias, and all the constantly monitored aspects, are embedded within the mobile platform. Screening and collection of demographic data will be performed during a face-to-face meeting.

### Screening and demographics

All participants will be asked to provide data at the beginning of their application regarding their age, sex, education level, living arrangements, marital status, income source (i.e., employed/unemployed, student, etc.), the total number of hours per week spent on sedentary behaviors, weight (kg), and BMI (kg/m^2^). Other recorded data includes dieting status, pregnancy status, menopause status, current medical and psychological treatment, and dietary requirements. As part of the detailed face-to-face screening, former and current medical and psychiatric history (via the Structured Clinical Interview DSM-IV; [49]) will also be assessed.

### Pre, post, and follow-up evaluation of the primary and secondary outcomes

#### Primary outcomes

Maladaptive thoughts related to eating and body weight will be assessed at T1, T2, and T3 via the Eating Disorders Beliefs Questionnaire (EDBQ; [50]). The 32-item EDBQ consists of four subscales: (1) negative self-beliefs, (2) weight and shape as a means to acceptance by others, (3) weight and shape as a means to self-acceptance, and (4) control over eating. The instrument has good reliability with a Cronbach’s alpha of 0.93, 0.94, 0.88, and 0.86, for the mentioned subscales, respectively, and good construct validity.

Eating behaviors will be assessed via the Dutch Eating Behavior Questionnaire (DEBQ; [51]). The DEBQ has 33 items clustered in three subscales: (1) restrained eating, (2) emotional eating, and (3) external eating. The subscales of the DEBQ have a high internal consistency and factorial validity with a Cronbach’s alpha of 0.95, 0.94, and 0.80 for the restrained, emotional, and external eating subscales, respectively.

Food cravings will be evaluated via the Trait and State Food Cravings Questionnaires (FCS-S, FCS-T; [52]). The FCS has good psychometric properties and assesses constructs as follows: desire to eat, anticipation of positive reinforcement, anticipation of relief from negative states and post-eating mood, lack of control over eating and craving as a physiological state. The FCS-S consists of 15 items, while the FCS-T consists of 39 items. Both questionnaires have good psychometric properties (Cronbach’s alpha of 0.97 for the FCS-T and 0.94 for the FCS-S).

The Binge Eating Scale (BES; [53]) will be used in order to assess the presence of binge-eating behaviors. The BES consists of 16 items, describing behaviors, emotions, and cognitions surrounding a binge episode (e.g., guilt, fear of not being able to stop eating). The BES was demonstrated to have an excellent internal consistency (Cronbach’s alpha of 0.87; [54]).

In order to assess attentional biases towards healthy and unhealthy food, a modified dot-probe task will be employed [55]. Each trial begins with displaying a fixation cross in the center of the screen for 500 ms, followed by displaying a picture pair for another 500 ms. The relevant pairs consist of pictures of healthy and unhealthy food items and the control/neutral pairs consist of animal pictures. The pictures are displayed on the left and right of the screen, at equal distances from the center. After the pictures disappear, a probe stimulus appears, replacing one of the two images. Participants have to determine as fast as possible whether the probe stimulus replaces the picture on the left or on the right side of the screen by pressing a corresponding key. The order in which the picture pairs are presented will be randomized for all participants.

Attentional bias scores will be calculated by subtracting the mean reaction time to the probes replacing healthy food pictures from the mean reaction time to the probes replacing unhealthy food pictures. Positive scores are indicative of an attentional bias towards healthy food items, while negative scores are indicative of an attentional bias towards unhealthy ones. Reaction times for all the trials will be used to assess attentional biases. Incorrect responses and outlier reaction times will be removed from the analysis.

#### Secondary outcomes

Levels of depression, anxiety, and stress will be evaluated with the Depression, Anxiety, and Stress Scales (DASS; [56]). DASS is a set of three self-report scales designed to measure the negative emotional states of depression, anxiety, and stress and has good psychometric properties (Cronbach’s alpha = 0.96; 0.89; 0.93 for the depression, anxiety, and stress scales, respectively).

The general mood will be evaluated with the Positive and Negative Affect Schedule – Short Form (PANAS-SF; [57]). PANAS has two mood subscales, the positive affect subscale, and the negative affect subscale and has good psychometric properties (Cronbach’s alpha = 0.86 to 0.90).

Weight (kg) and BMI (kg/m^2^) measures will be self-reported via the SIGMA application. Furthermore, physical activity will be assessed via the SIGMA app’s incorporated pedometer and a mean daily step count will be extracted for baseline, post intervention, and follow-up.

#### Constantly monitored aspects

A special feature of the monitoring module is the *ABC diary*. The *ABC diary* is meant to be filled in (1) at the end of the day after an unplanned meal or after an impulsive eating episode took place or (2) in problematic situations, while still contemplating yielding to eating urges. Hence, the *ABC diary* monitoring tool has two features, as detailed below.

When filled in at the end of the day, the *ABC diary* has an awareness role as users can notice what self-reported emotions, cravings, and sabotaging thoughts preceded and ensued their eating behavior. As such, users can choose from a list of emotions, cravings, and beliefs and rate their intensity, or fill in some of their own. An automatically generated graph indicates whether a change in time occurred in any of these variables and pinpoints to triggering/problematic situations.

When filled in problematic situations, the *ABC diary* has a preventive role. In addition to recording emotions, cravings, and sabotaging thoughts, the *ABC diary* monitoring tool provides healthy alternative ways of thinking/coping tips or allows the user to write some personally motivational healthy statements. If alternative ways of thinking do not decrease the urge or desire to eat, then the user is redirected to the Crisis and Relapse prevention module in an effort to reduce the relapse rate.

The healthy habit points system is another constantly monitored aspect. Within the SIGMA application, there are three possible sources of earning points: (1) the explicit cognitive-behavioral intervention (SIGMAe), (b) the implicit attention-training intervention (SIGMAi), and (c) the pedometer. In SIGMAi and SIGMAe awarded points vary from 0 to 8 healthy habit points. Regarding the pedometer, the points received by the user each day will be proportional to the number of steps taken (i.e., 5 points for 5000 steps, 8 points for 8000 steps, etc.). Furthermore, a calorie counter is also available to keep track of daily calorie consumption. However, the application does not encourage a fixation on calorie counting [45]. Hence, no points are earned for its usage. Overall, the points gathered from SIGMAe, SIGMAi, and the pedometer will be assessed separately, as well as pooled into a total score indicative of overall learning and adherence to the intervention.

### Data analysis

To test the efficacy of the SIGMA intervention against the attention placebo control group a 2 (group: SIGMA intervention group versus attention placebo group) × 3 (time: pre vs. post vs. follow-up) general linear mixed model will be used with regard to the primary and secondary outcomes. Separate analyses will be performed for the “intent-to-treat” and the completer sample. The intent-to-treat principle will be employed [58] with the last observation carried forward method. The Bonferroni-Holm correction will be used to adjust for multiple comparisons. Overall, with a sample of at least 74 participants, the trial will be powered to identify at least a medium effect size (i.e., Cohen’s *d* = 0.50).

App usage data and user activity will be inspected by contrasting; for example, the participants’ self-reported activity with their actual app usage as recorded in the database. This will inform us about any impediments to user adherence, as well as particular usage patterns.

### Ethics

The current trial protocol was approved by the Ethics Committee of the Babeș-Bolyai University and the University of Bucharest. Several measures will be taken in order to protect the participants’ wellbeing and identity as follows.

Firstly, according to the exclusion criteria, participants suffering from psychiatric conditions (i.e., including clinical eating disorders), as well as other serious health conditions that are incompatible with undertaking a weight-loss regimen, will not be considered for inclusion and will immediately be referred to healthcare professionals.

Secondly, if participants’ physical or emotional condition deteriorates during the trial, they will immediately be excluded from the trial and will similarly be referred to appropriate practitioners. Participants will be informed that participation is voluntary and that they may discontinue the intervention at their free will. Any adverse events and other unintended effects of trial interventions or trial conduct will be addressed by the project coordinator (IRP).

With respect to data protection, in addition to user authentication via username and password, all data protection issues will be covered by (1) having the locally stored data written in binary files that are difficult to alter, (2) by ensuring a secure HTTPS data transfer protocol, (3) by having a server authentication of the researchers, and (4) by using user aliases accessed by authorized personnel only. Furthermore, the customized feedback reports of each participant will only be available to themselves and protected by means of unique usernames and passwords.

## Discussion

The SIGMA study is a randomized, placebo-controlled trial entailed to test the efficacy of the SIGMA mHealth intervention against an attention placebo control group. The SIGMA mHealth intervention combines a portable, serious game interface with evidence-based theoretical models and up-to-date cognitive and behavioral principles for weight management. Furthermore, it targets overweight young adults with maladaptive eating habits that are at risk for obesity. Hence, the SIGMA intervention also incorporates attributes that are specific for preventive interventions and is, to our knowledge, the first evidence-based serious game for weight management. In addition, there are no RCTs that examined the efficacy of mHealth interventions in overweight and/or obese young adults, and even more so on individuals with maladaptive eating patterns.

Despite its advantages, the SIGMA intervention is subject to several limitations. Firstly, the data concerning maladaptive eating patterns, as well as weight and BMI, will be collected by means of self-report. Therefore, being susceptible to distortions resulting from social desirability effects. However, many of these outcomes require subjective judgments, thus self-reports are inevitable. When possible, we try to supplement our subjective measures with more objective ones, such as reaction times or daily step counts. Secondly, the decision to allow participants to follow a diet of their own choice potentially adds a source of variability in the results but only with regard to the secondary outcomes. However, as evidenced in a recent comprehensive meta-analysis, the ability to freely choose one’s diet promotes adherence to the weight-loss program [59]. Moreover, recent results indicate that different types of calorie-restrictive diets are equally effective as long as they are appropriately followed (e.g., [59, 60]). This approach is highly relevant for self-help programs enabling weight-loss monitoring in a self-guided matter. One example in this respect is the “Think Slim” intervention [61].

Overall, the SIGMA intervention has several noteworthy contributions, as exemplified in the following. *Firstly*, it relies on evidence-based practices for weight management in individuals challenged by maladaptive eating habits [36]. *Secondl*y, it not only addresses maladaptive eating patterns, such as emotional eating, food craving, binge eating, but it also addresses their cause as embodied by maladaptive cognitive styles. *Thirdly*, it aims at curbing the elevated attrition rates specific for weight management programs [62] by employing a gamified approach that is both interactive and engaging. *Fourthly*, SIGMA proposes an element of novelty among mHealth interventions for weight management in the sense that it includes cognitive techniques alongside standard behavioral techniques for long-lasting lifestyle changes and weight maintenance [7].

Given the increase in rates of obesity, we conclude that the SIGMA intervention may provide a cost-effective (i.e., always available) and preventive self-help tool for young overweight adults with maladaptive eating habits.

### Trial status

Recruiting

## Additional file

Additional file 1: SPIRIT 2013 Checklist: recommended items to address in a clinical trial protocol and related documents. (DOC 123 kb)
